# Advances in targeted therapy for acute myeloid leukemia

**DOI:** 10.1186/s40364-020-00196-2

**Published:** 2020-05-20

**Authors:** Jifeng Yu, Peter Y. Z. Jiang, Hao Sun, Xia Zhang, Zhongxing Jiang, Yingmei Li, Yongping Song

**Affiliations:** 1 The First Affiliated Hospital of Zhengzhou University, #1 East Jianshe Road, Zhengzhou, 450052 China; 2 Academy of Medical and Pharmaceutical Sciences of Zhengzhou University, #1 East Jianshe Road, Zhengzhou, 450052 China; 3 Department of Hematology and Oncology, The Everett Clinic and Providence Regional Cancer Partnership, 1717 13th Street, Everett, WA 98201 USA; 4 The Affiliated Cancer Hospital of Zhengzhou University and Henan Cancer Hospital, 127 Dongming Road, Zhengzhou, 450008 China

**Keywords:** Targeted therapy, Gene mutation, Acute myeloid leukemia (AML)

## Abstract

Acute myeloid leukemia (AML) is a clonal malignancy characterized by genetic heterogeneity due to recurrent gene mutations. Treatment with cytotoxic chemotherapy has been the standard of care for more than half of a century. Although much progress has been made toward improving treatment related mortality rate in the past few decades, long term overall survival has stagnated. Exciting developments of gene mutation-targeted therapeutic agents are now changing the landscape in AML treatment. New agents offer more clinical options for patients and also confer a more promising outcome. Since Midostaurin, a FLT3 inhibitor, was first approved by US FDA in 2017 as the first gene mutation-targeted therapeutic agent, an array of new gene mutation-targeted agents are now available for AML treatment. In this review, we will summarize the recent advances in gene mutation-targeted therapies for patients with AML.

## Introduction

Acute myeloid leukemia (AML) is a clonal malignancy originating from hematopoietic stem cells, characterized by heterogeneous chromosomal abnormalities, recurrent gene mutations, epigenetic modifications affecting chromatin structure, and microRNAs deregulations. Genomic heterogeneity, patients’ individual variability, and gene mutations are few major obstacles among the many factors that impact treatment efficacy for AML patients [1, 2].

Different strategies have been used to treat various types of cancer in preclinical models [3, 4]. Traditional chemotherapy using cytotoxic agents in AML treatment had been the main modality for decades. New molecular techniques, however, such as next-generation sequencing (NGS) identifying important genetic alterations, have paved the path for new drug development targeting those specific gene mutations. Since the past few years, the state-of-the-art treatment for AML has evolved rapidly: cytogenetic and molecular interactions being more individualized, the state of minimal residual disease (MRD) detected by flow cytometry and NGS, and incorporation of gene mutation-targeted novel therapies. In combination with precise clinical diagnosis and detailed risk stratification, gene mutation-targeted new drug therapies have made breakthrough and promising progresses for patients with AML [5, 6].

In April 2017, the US Food and Drug Administration (FDA) approved Midostaurin, a FMS-like tyrosine kinase 3 (FLT3) inhibitor, for AML patients with FLT3 mutations. Midostaurin is the first tyrosine kinase inhibitor (TKI) approved for AML; and it is also the first drug approved in a mutation-specific and non–acute promyelocytic leukemia (APL) subtype. Since then, many gene mutation-targeted therapies for AML have emerged, such as Enasidenib, an isocitrate dehydrogenase (IDH)2 inhibitor, for relapsed/refractory (R/R) AML with IDH2 mutations [7–9]. The “one-size-fits-all” cytotoxic chemotherapy regimen will soon be enhanced or replaced by more specific targeted treatment in AML.

Targeted therapy in AML can be divided into 3 groups: Group 1: agents that act on oncogenic effectors of recurrent AML associated mutations, which include FLT3 and IDH inhibitors. Group 2: agents that act on disrupting key cell metabolic or maintenance pathways without directly damaging DNA or its repair. These include epigenetic modifiers and agents that directly target apoptosis. Group 3: agents that act by targeted delivery of cytotoxic agents, such as ADCs [10]. In this review article, we will focus on the advances in the gene mutation-targeted agents, including FLT3 inhibitors, IDH inhibitors and Smoothened (SMO) inhibitors.

### FLT3 inhibitors

FLT3 is a transmembrane ligand-activated receptor tyrosine kinase (RTK) which plays an important role in the early stages of both myeloid and lymphoid lineage development. FLT3 ligand binds and activates FLT3 through various signaling pathways, such as PI3K, RAS, and STAT5 [11]. FLT3 mutations are found in approximately 30–35% of newly diagnosed AML cases with either internal tandem duplications (FLT3-ITD) within the juxtamembrane domain coding region (exons 14 and 15, [12]) or missense mutations in the tyrosine kinase domain (FLT3-TKD) in the activation loop (exon20) [13]. FLT3-ITD and FLT3-TKD type mutations occur in about 25% and 7–10% of AML patients, respectively [14–17]. Data have suggested that there are racial and ethnic disparities in genetic alteration between Caucasian and Eastern Asian population. Lower proportion of FLT3-ITD mutation and more AML patients with core binding factor leukemia have been found in Eastern Asian cohorts [18]. FLT3-ITD mutation had been considered as a negative prognostic marker, used for AML risk stratification and disease monitoring via MRD, with the clinical importance of early detection at diagnosis and again at relapse [2].

As progresses have been made in understanding the mechanism of FLT3 gene mutation, TKI agents have been developed by targeting different points of the ATP binding site in the intracellular domain of the FLT3 RTK: Type 1 inhibitors, which include Sunitinib, Lestaurtinib, Midostaurin, Crenolanib, and Gilteritinib [19], bind to the RTK ATP-binding site in the active conformation and the inactive state; Type 2 inhibitors, which include Sorafenib, Quizartinib and Ponatinib [19, 20], bind to the hydrophobic region in juxtaposition to ATP-binding domain when RTK is in the inactive state and prevent receptor activation.

#### Midostaurin

Midostaurin was approved by the US FDA for AML induction and consolidation based on the RATIFY trial, which took 13 years to complete [7]. The RATIFY trial was the first large multicenter study investigating the addition of Midostaurin to induction and consolidation and continued as maintenance therapy for 1 year in patients not proceeding to allogeneic transplant [9, 21, 22]. Patients with FLT3 mutations, either ITD or TKD, had a 4-year survival of 51.4% on Midostaurin versus 44.2% on placebo (*P* = .0074), and the benefit was most pronounced in Nucleophosmin 1 (NPM1) wt and FLT3^high^ patients [9]. In another study, Midostaurin was added to intensive induction chemotherapy, consolidation and continued as maintenance in FLT3-ITD AML patients complete remission (CR) plus complete remission with incomplete hematologic recovery (CRi) after induction therapy was observed in 76.4% patients. Event free survival (EFS) and overall survival (OS) at 2 years were 39 and 34% in younger and 53 and 46% in older patients, respectively. Propensity score-weighted analysis revealed a significant improvement of EFS by Midostaurin overall and in older patients [23].

In a new retrospective exploratory study, multivariate Cox model for OS using allogeneic hematopoietic stem cell transplantation (allo-HSCT) in first complete remission (CR1) as a time-dependent variable revealed treatment with Midostaurin, allo-HSCT, European Leukemia Net (ELN) favorable-risk group, and lower WBC counts as significant favorable factors. There was a consistent beneficial effect of Midostaurin across ELN risk groups [24]. Midostaurin has been recommended as frontline therapy for the FLT3 gene mutated AML patients with either FLT3-ITD or FLT3-TKD [19, 25]. It’s also been proved cost-effectiveness when Midostaurin was combined with standard chemotherapy in the treatment of newly diagnosed FLT3-mutated AML patients [26].

Midostaurin is among the least potent FLT3 inhibitors. More potent FLT3 inhibitors are Gilteritinib, Quizartinib, and Crenolanib [20]. Early phase trials combining these newer generation FLT3 TKIs with 7 + 3 induction chemotherapy in the frontline setting have been reported recently with meaningfully higher response rate [25].

#### Gilteritinib

Gilteritinib is an orally available small molecule receptor TKI for the treatment of AML harboring FLT3 mutations. Gilteritinib inhibits FLT3 signaling in cells expressing FLT3-ITD, TKD mutation FLT3-D835Y and the double mutant FLT3-ITD-D835Y, thereby inducing apoptosis. Gilteritinib also binds to and inhibits the wild-type and mutated forms of anaplastic lymphoma kinase (ALK), resulting in reduced tumor cell proliferation in cancer cell types that overexpress the mutation [27, 28].

In a phase 3 trial with R/R FLT3-mutated AML, the median OS for the group with single agent Gilteritinib was significantly longer than that of the group with chemotherapy (9.3 months vs. 5.6 months). The median EFS was 2.8 months in the Gilteritinib group and 0.7 months in the chemotherapy group. The percentage of patients who achieved complete remission with full or partial hematologic recovery was 34.0% in the Gilteritinib group and 15.3% in the chemotherapy group. Gilteritinib resulted in significantly longer survival and higher percentages of CR than salvage chemotherapy among patients with R/R FLT3-mutated AML [29]. These findings confirm the superior efficacy of Gilteritinib over chemotherapy for patients with FLT3-mutant AML. Currently, multiple clinical trials are ongoing to evaluate the combination of Gilteritinib with other agents and regimens [25, 30]. These clinical studies supported Gilteritinib’s approval by US FDA in 2018 as the new standard therapy for R/R FLT3-mutated AML [31].

#### Sunitinib

Sunitinib (SU11248) is a small-molecule FLT3 inhibitor with selectivity for FLT3 and others, such as platelet-derived growth factor receptors (PDGFR), vascular endothelial growth factor receptor (VEGFR1) 1, VEGFR2, and KIT [32]. It has both direct anti-tumor and anti-angiogenic properties [33]. One study found that the signal transducer and activator of transcription 5 (STAT5) phosphorylation in patients with FLT3-ITD was also reduced [34]. Furthermore, Sunitinib induces G1 phase arrest, increases pro-apoptotic molecule expression, and decreases anti-apoptotic molecule expression in AML cells [35].

Intriguingly, Sunitinib shows synergistic effects with Cytarabine or Daunorubicin in inhibiting proliferation and survival of primary AML myeloblasts expressing mutant FLT3-ITD, FLT3-D835V, or FLT3-WT [36]. Several early clinical trials evaluating Sunitinib in combination with chemotherapy had shown some promising results in phase I/II clinical trials [37, 38], however, due to high incidence of adverse effects such as blood and lymphatic system disorders, cardiac disorders, gastrointestinal disorders and others, no clinical trials in the realm of hematological malignancy are actively going on. Sunitinib has been approved and widely used for treatment of several solid tumors, such as renal cell cancer, gastrointestinal stromal cell tumor, and neuroendocrine tumors.

#### Lestaurtinib

Lestaurtinib (CEP-701) is a multi-targeted TKI that potently inhibits FLT3 tyrosine kinase and induces hematological remission in AML patients harboring FLT3-ITD. However, the majority of patients in clinical trials developed resistance to CEP-701. Although restoration of SHP-1 expression induces sensitivity towards CEP-701 and could serve as a target in the treatment of AML [39], Lestaurtinib failed to demonstrate any overall clinical benefit in a phase III trial when combined with intensive chemotherapy in patients with newly diagnosed FLT3-ITD-mutated AML [25, 40, 41].

#### Crenolanib

Crenolanib, a potent type I pan-FLT3 inhibitor, is effective against both ITD and resistance-conferring TKD mutations. While Crenolanib monotherapy has demonstrated clinical benefit in heavily pretreated R/R AML patients, responses are transient and relapse eventually occurs [42]. Study on the mechanisms of Crenolanib resistance has been done by performing whole exome sequencing of AML patient samples before and after Crenolanib treatment. Unlike other FLT3 inhibitors, Crenolanib does not induce FLT3 secondary mutations, and mutations of the FLT3 gatekeeper residue are infrequent. Instead, mutations of NRAS and IDH2 arise mostly as FLT3-independent subclones. Meanwhile TET2 and IDH1 predominantly co-occur with FLT3-mutant clones and are enriched in Crenolanib poor-responders. The other patients have exhibited post-Crenolanib expansion of mutations associated with epigenetic regulators, transcription factors, and cohesion factors, suggesting diverse genetic/epigenetic mechanisms of Crenolanib resistance. Drug combinations in experimental models can restore Crenolanib sensitivity [42].

Crenolanib was well tolerated in a phase II trial in combination with 7 + 3 induction therapy in newly diagnosed FLT3-mutated AM patients. Addition of Crenolanib to induction chemotherapy in patients with concurrent FLT3 and other mutations, such as NPM1, DNA methyltransferase 3A (DNMT3A), Runt-related transcription factor 1 (RUNX1), or Wilms’ tumour 1 (WT1), can overcome the poor prognostic implication of adverse mutations co-occurring with mutated FLT3 [12].

Ongoing clinical trials are assessing the efficacy of Crenolanib in combination with intensive salvage chemotherapy for patients with R/R FLT3 mutant AML (NCT2626338). Incorporation of Crenolanib into frontline intensive chemotherapy regimens have resulted in higher response rates and may eventually replace Midostaurin in the upfront setting [43]. Currently, there are 6 registered on-going clinical studies of Crenolanib for AML patients as of February 2020 (Table 1).

**Table 1:** Current clinical trials of Crenolanib for leukemia patients

ClinicalTrials. gov Identifier	Phase	Enrollment Number	Disease Conditons	Status	Lead Institution/Location
NCT02298166	3	276	AML	Active, not recruiting	Ulm University Hospital, Germany
NCT02400255	2	48	AML	Active, recruiting	MD Anderson Cancer Center, USA
NCT02400281	1,2	88	AML	Active, not recruiting	MD Anderson Cancer Center, USA
NCT02283177	2	48	AML with FLT3 Mutations	Active, not recruiting	City of Hope, USA
NCT03250338	2	322	R/R AML with FLT3 mutations	Active, recruiting	City of Hope, USA
NCT03258931	3	510	FLT3 mutated AML	Active, recruiting	City of Hope, USA

#### Quizartinib

Quizartinib is a potent and selective type 2 FLT3 inhibitor and has been used as an effective therapy for patients with FLT3-ITD AML. Quizartinib inhibits FLT3 thereby dampen oncogenic drive, leading to apoptosis of tumor cells. Phase 1 study demonstrated efficacy when combined with induction chemotherapy, and when used as monotherapy for maintenance therapy in AML patients after allo-HSCT [44, 45]. Another phase 1 multicenter dose-escalation study assessing the safety/tolerability of Quizartinib maintenance post-HSCT in FLT3^+^ AML demonstrated the safety, with promising results in the first 13 patients treated and no increase in graft versus host disease (GvHD) [46]. A phase 2 study demonstrated the potency and efficacy of Quizartinib amongst FLT3^+^ R/R AML patients within 1 year of induction therapy, or who had undergone salvage chemotherapy, or allo-HSCT [47, 48].

A phase III QUANTUM-R study further substantiated Quizartinib’s efficacy. Quizartinib could be considered a new standard of care for patients with rapidly proliferative disease and very poor prognosis [49]. Multiple clinical trials have proved its efficacy in R/R AML with FLT3-ITD mutation. Quizartinib resistance has been observed in clinical treatment. Further clinical studies are ongoing aiming to reduce toxicity, increase efficacy by combining with a targeted drug for RUNX1, and identify a predictive response biomarker in patients [47, 48, 50].

Strategies on combining Quizartinib with other TKI agents like Crenolinib, PIM kinase, and MEK inhibitors should be further explored [51]. When managing patients on Quizartinib, some special situations need to be considered for adequate scheduling and tolerability, bridging to allo-HSCT, and durable remission on maintenance therapy [52]. Currently, there are 15 registered on-going clinical studies of Quizartinib for leukemia patients as of February 2020 (Table 2).

**Table 2:** Current clinical trials of Quizartinib for leukemia patients

ClinicalTrials. gov Identifier	Phase	Enrollment Number	Disease Conditons	Status	Lead Institution/Location
NCT04107727	2	281	AML	Active, recruiting	Complejo Hospitalario Universitario de A Coruña, Spain
NCT03552029	1	156	AML	Active, recruiting	Ronald Reagan Medical Center, UCLA, USA
NCT03735875	1,2	32	AML with FLT3/ITD mutation R/R AML	Active, recruiting	M D Anderson Cancer Center, USA
NCT03661307	1,2	52	AML with TP53 gene mutation/deletion R/R AML High risk, R/R MDS	Active, recruiting	M D Anderson Cancer Center, USA
NCT02668653	3	539	AML	Avtive, not recuiting	University of Florida (UF) Health Shands Hospital, USA
NCT04112589	1,2	80	AML	Active, recruiting	Centro Hospitalar e Universitário de Coimbra, Portuga
NCT03793478	1,2	65	AML	Active, recruiting	Loma Linda University Cancer Center, USA
NCT03723681	1	18	AML	Active, recruiting	Institute of Hematology and Blood Diseases Hospital CAMS, China
NCT04128748	1,2	52	R/R AML High risk, R/R MDS	Active, not recruiting	M D Anderson Cancer Center, USA
NCT01892371	1,2	200	R/R AML with FLT3 mutation High risk, R/R MDS R/R CML	Active, not recruiting	M D Anderson Cancer Center, USA
NCT04047641	2	86	AML R/R AML High risk, R/R MDS	Active, recruiting	M D Anderson Cancer Center, USA
NCT04209725	2	34	AML	Active, not recruiting	Colorado Blood Cancer Institute, USA
NCT02039726	3	367	AML	Active, not recruiting	City of Hope, USA
NCT03135054	2	40	AML with FLT3-ITD mutation	Active, recruiting	The University of Hong Kong, Hong Kong
NCT03989713	2	80	AML R/R AML	Active, not recruiting	University Hospital Heidelberg, Germany

#### Sorafenib

The multi-kinase inhibitor Sorafenib has demonstrated modest efficacy in FLT3^+^ AML as monotherapy. Sorafenib’s effect against AML is similar to that of Sunitinib [33]. However, resistance limited its use as a single agent [20, 53]. In combination with standard chemotherapy in patients under the age of 60, Sorafenib prolongs survival with modestly increased toxicity [54]. The survival benefit is less clear in patients over the age of 60 when added to standard induction chemotherapy [55, 56]. Sorafenib maintenance following allo-HSCT resulted in improved OS and EFS [57–59]. Sorafenib before transplantation, Sorafenib maintenance after transplantation, and their combined application all could improve the outcomes for patients with FLT3-ITD AML. Sorafenib’s effect on induction therapy and maintenance following allo-HSCT lead to superior 3-year EFS and 3-year OS as induction, re-induction therapy, or post-transplant as maintenance therapy when compared to no Sorafenib use. There was superior LFS with any use of Sorafenib with most benefit seeing in the group receiving both pre-transplant and post-transplant [60]. A phase 2 clinical trial showed Sorafenib and Omacetaxine Mepesuccinate as a safe and effective treatment for AML with FLT3-ITD mutation [61].

Allo-HSCT plus Sorafenib maintenance was an effective strategy to improve recurrence free survival and decrease relapse probability in FLT3-ITD AML patients. It had benefits to AML patients regardless of ITD mutant ratio, and to those with long ITD length instead of the short ITD length [62]. A prospective study of patients with FLT3-ITD AML undergoing allo-HSCT was conducted to evaluate the safety, tolerability, and outcome of Sorafenib administered peritransplant. Sorafenib dosing was individualized in the post-transplantation setting according to patient tolerability. Results indicate that Sorafenib is effective in vivo FLT3 inhibition and yields encouraging survival results [63].

Another study showed Sorafenib plus intensive chemotherapy improves survival in patients with newly diagnosed FLT3-ITD mutated AML regardless of whether they undergo allo-HSCT [64]. Addition of Sorafenib to chemotherapy not only nullifies the negative prognostic impact of higher allele burden, but also improves outcome of FLT3-ITD mutated AML patients regardless of the allele burden [65]. Sorafenib therapy is associated with improved outcomes for FLT3-ITD AML relapsing after allo-HSCT. Sorafenib combined with chemotherapy followed by donor lymphocyte infusion reveals an optimal efficacy [66]. Combination of Sorafenib with hypomethylating agents (azacitidine or decitabine) has resulted in high response rates in patients with FLT3 mutant AML inappropriate for intensive chemotherapy. FLT3 inhibitors are being explored in combination with other targeted agents [67]. Sorafenib has been approved and widely used in solid tumors, such as renal cell cancer, hepatocellular cancer, etc. [68, 69].

#### Ponatinib

As the second generation TKI, Ponatinib has been indicated for patients with TKI resistant chronic myeloid leukemia (CML) [70]. Recent results showed Ponatinib also comprises a high capability to inhibit constitutively activated FLT3. Ponatinib is able to overcome resistance to other TKI (e.g., Sorafenib) if it is conferred by additional point mutations of FLT3-ITD. It represents a promising compound in FLT3-ITD positive AML as well [71]. No systematic investigation of Ponatinib in AML patients has yet presented [72]. As of February 2020, there are 10 on-going clinical studies for Ponatinib mainly in CML patients from different research centers (Table 3).

**Table 3:** Current clinical trials of Ponatinib for leukemia patients

ClinicalTrials. gov Identifier	Phase	Enrollment Number	Disease Conditons	Status	Lead Institution/Location
NCT02398825	2	78	CML	Active, recruiting	Azienda Ospedaliero Universitaria Ospedali Riuniti Umberto, Italy
NCT04048564	Observational	150	CML	Active, recruiting	CHU SUD Reunion GHSR, France
NCT03807479	2	54	CML	Active, recruiting	University Hospital RWTH Aachen, Germany
NCT02627677	3	44	CML	Active, not recruiting	Cliniques Universitaire Saint Luc, Belgium
NCT01641107	2	44	BCR-ABL+ ALL	Active, not recruiting	S.O.C. di Ematologia, Italy
NCT03934372	1,2	60	AML, ALL All Phase CML Solid Tumors	Active, recruiting	UZ Gent, Belgium
NCT03933852	Observational	100	CML	Active, recruiting	University Hospital Jena, Germany
NCT03678454	Observational	125	CML Ph + ALL	Active, recruiting	ZNA Stuyvenberg, Belgium
NCT03147612	2	60	Accelerated Phase CML BCR-ABL1+ R/R ALL	Active, recruiting	M D Anderson Cancer Center, USA
NCT01746836	2	50	Chronic Phase CML BCRABL1+ Recurrent CML BCRABL1+	Active, not recruiting	M D Anderson Cancer Center, USA

### IDH inhibitors

Mutations in the IDH gene, specifically R132 in IDH1, R140 and R172 in IDH2, are substrates for targeted therapy [73]. IDH1 and IDH2 are commonly mutated in cytogenetically normal AML (IDH1 6–16%, IDH2 8–19%). They impart a critical role in cellular metabolism by catalyzing the conversion of alpha-ketoglutarate to the oncometabolite R enantiomer of 2 hydroxyglutarate (R-2HG) [7, 73–75]. R-2HG inhibits cellular differentiation and promotes proliferation via TET2 inhibition and downstream effects of demethylation in vitro. It has a pivotal role of IDH mutations in leukemogenesis [76, 77]. Though currently not a component of the ELN guidelines for prognostication, IDH1/2 assessment should now be routinely done in AML patients, due to the availability of targeted therapy with the IDH1 and IDH2 inhibitors, Ivosidenib and Enasidenib [2].

IDH inhibitors (IDH-i) are used in patients with AML who have mutations in either IDH1 or IDH2 genes, causing abnormal maturation patterns in white blood cells, thus leading to leukemia [78]. Two targeted IDH-i: Ivosidenib and Enasidenib, blocking the proteins IDH1 and IDH2, respectively. The inhibition leads the leukemic cells to normal maturation and differentiation, thereby reducing immature blast counts and increasing the percentage of mature myoblasts [79, 80]. A safety concern with IDH-i is the possible side effect known as differentiation syndrome, the release of inflammatory cytokines from cancerous promyelocytes, referred to “cytokine storm”. Cytokine storm is serious and potentially fatal but can be reversed by stopping the offending agent [81].

#### Ivosidenib

IDH1 inhibitor Ivosidenib demonstrated overall safety and efficacy amongst patients with IDH1-mutated R/R AML leading to FDA approval [8]. In a phase 1 clinical trial, Ivosidenib monotherapy was well tolerated and induced durable remissions and transfusion independence in patients with newly diagnosed AML. IDH1 mutation clearance was seen in 9/14 patients achieving CR + CRh (5/10 CR, 4/4 CRh) [82]. Ivosidenib benefits a group of patients with poor prognosis and limited options. Although reports have revealed acquired resistance for these mutant IDH inhibitors, combination treatment can overcome this problem [83, 84]. Interestingly, amongst IDH1-mutated myelodysplastic syndrome (MDS) patients who were refractory to therapy with hypomethylating agents, Ivosidenib appeared to have a substantial efficacy, though the subgroup was small (*n* = 12) [8]. Accumulating data has indicated that targeted therapy using Ivosidenib may represent an encouraging therapeutic option in patients with acute undifferentiated leukemia and IDH1 mutations [85]. Currently, there are 10 registered active clinical studies for Ivosidenib in AML patients at different research centers (Table 4).

**Table 4:** Current clinical trials of Ivosidenib for leukemia patients

ClinicalTrials. gov Identifier	Phase	Enrollment Number	Disease Conditons	Status	Lead Institution/Location
NCT04250051	1	25	R/R AML R/R MDS R/R MPN	Not yet recruiting	Northwestern University, USA
NCT03839771	3	968	AML MDS EB-2	Active, recruiting	Erasmus MC, Netherland
NCT03173248	3	392	Newly Diagnosed AML AML Arising From MDS	Active, recruiting	City of Hope, USA
NCT02677922	1,2	131	AML	Active, not recruiting	City of Hope, USA
NCT04176393	1	30	R/R AML	Active, recruiting	Institute of Hematology and Blood Diseases Hospital, CAMS, China
NCT02632708	1	153	Newly Diagnosed AML AML Arising From MDS, AHD AML Arising After Exposure to Genotoxic Injury	Active, not recruiting	City of Hope, USA
NCT04044209	2	45	MDS AML	Active, not recruiting	Yale Cancer Center, USA
NCT03471260	1,2	48	High risk MDS MPN R/R AML	Active, recruiting	Northwestern Medicine Cancer Center Delnor, USA
NCT03503409	2	68	MDS AML	Active, recruiting	CH Angers, France
NCT02074839	1	291	R/R AML Other IDH1 mutated+ Hematologic Malignancies MDS	Active, recruiting	Birmingham, USA

#### Enasidenib

Enasidenib is a FDA approved agent in the treatment of R/R AML. As the first-in-class mutant IDH2 inhibitor, Enasidenib has demonstrated safety and efficacy in phase 1/2 dose escalation and dose-expansion study [7]. Enasidenib was well tolerated and induced molecular remissions and hematologic responses in patients with AML for whom prior treatments had failed [86]. In clinical trials, Enasidenib has demonstrated remarkable activity in patients with mutated IDH2 [87]. Enasidenib has shown clinical activity in patients with R/R AML. Inducing differentiation of myeloblasts, not cytotoxicity, seems to drive the clinical efficacy of Enasidenib [8]. Recent research results have demonstrated Enasidenib motivated human erythroid differentiation independent of IDH2 and proved as a promising therapeutic agent for improving anemia. These results provided the basis for clinical trials using Enasidenib to decrease transfusion dependence in a wide array of clinical contexts [88]. Enasidenib is currently approved for the treatment of R/R AML at a dose of 100 mg oral daily. Study demonstrated that Enasidenib induces durable remissions in older patients with newly diagnosed AML. Oral, outpatient targeted treatment with Enasidenib may benefit older adults with newly diagnosed IDH2-mutant AML who are not candidates for cytotoxic regimens [89].

Older patients with AML are less likely to benefit from intensive chemotherapy. Instead, they benefit more from lower-intensity therapies and from newly available targeted AML treatments including Enasidenib [90]. Furthermore, even in the absence of a conventional CR, lower-intensity therapies may provide meaningful clinical benefit, including improved survival and quality of life, by inducing hematologic improvement and transfusion independence [90]. Adverse effects including indirect hyperbilirubinemia and IDH inhibitor induced cytokine storm which can be life threatening and should be identified and treated promptly [80, 87].

. Given the fact that Enasidenib is highly specific inhibitor acting on an early stable mutation, it is conceivable that this agent could be of more value if used in combination with other targeted agents. The proper role for single mutation targeting in AML therapy needs to be carefully considered [91].

#### Olutasidenib

Another IHD inhibitor named Olutasidenib, originally designed for glioma and glioblastoma, has been explored for AML and MDS treatment recently. Preclinical data showed Olutasidenib as a potent, orally bioavailable, brain penetrant, and selective IDH1 inhibitor. It has excellent ADME/PK properties and reduces 2-hydroxyglutarate levels in IDH1 mutation xenograft tumor model [92]. The Phase 1 study (NCT02719574) assessed the safety, PK/PD, and clinical activity of in AML or MDS patients with IDH1 mutation. The Olutasidenib study results have shown favorable safety and clinical activity in IDH1 mutant R/R AML as single agent with ORR of 41% and in a combination regimen with ORR of 46%, and durable disease control. Olutasidenib induces deep responses with IDH1 mutation clearance in a subset of treated patients [93]. Meanwhile, Olutasidenib has shown favorable safety profile and clinical activity in IDH1 mutant MDS, with an ORR rate of 59% and durable disease control. Phase 2 trial is ongoing as single agent and in combination with Azacitidine [94]. This agent may have good potential for AML and MDS treatment; however more clinical researches need to be explored.

### Hedgehog signalling pathway inhibitors

The Hedgehog (Hh) signalling pathway is activated in many types of cancers including AML and naturally a promising target for therapeutic development. SMO plays very important role in the Hh signalling pathway and has been shown to be critical for acute leukemia disease progression. Approaches to inhibit Hh signalling for therapeutic benefit have focused primarily on SMO inhibitors. As a SMO antagonist, Glasdegib, an oral inhibitor of the Hedgehog signalling pathway, has been developed in clinical trials [95] in combination with standard chemotherapy for patients with AML or high-risk MDS [96, 97].

Glasdegib was approved in USA in November 2018 for use with low-dose cytarabine for treatment of patients with newly-diagnosed AML over age of 75 years or with comorbidities that precluding intensive induction chemotherapy. It is currently undergoing clinical development for use in other malignancies, including MDS, in various countries worldwide [98]. More recently, similar SMO inhibitors such as Vismodegib [99, 100], Sonidegib [100, 101] and Erismodegib [99, 102] are under-development in clinical trials for patients with AML and MDS, in combination with chemotherapies such as Azacytidine, a hypomethylating agent. Early results demonstrated promising responses in patients with AML and MDS. As of March 2020, there are 10 registered recruiting/completed clinical trials with Glasdegib, Vismodegib, Sonidegib and Erismodegib from the www.ClinicalTrials.gov website (Table 5).

**Table 5:** Current clinical trials of SMO inhibitors for leukemia patients

Agents	ClinicalTrials. gov Identifier	Phase	Enrollment Number	Disease Conditons	Status	Lead Institution/Location
Glasdegib	NCT03416179	3	720	AML	Recruiting	UCLA, USA
	NCT04051996	2	46	AML	Recruiting	Yale Cancer Center, USA
	NCT02367456	2	73	AML, MDS	Active, not recruiting	University of Alabama at Birmingham, USA
	NCT03390296	1/2	138	R/R AML	Recruiting	M D Anderson Cancer Center, USA
	NCT02038777	1	49	AML	Recruiting	Japanese Red Cross Nagoya First Hospital,Japan
	NCT01546038	2	255	AML	Completed	University of Alabama at Birmingham, USA
Vismodegib	NCT02593760	1	19	Myelofibrosis	Completed	Florida Cancer Specialists, USA
Sonidegib	NCT01826214	2	70	Acute Leukemia	Completed	Duke University Medical Center, USA
	NCT01456676	1	11	CML	Completed	Novartis Investigative Site, Canada
	NCT02129101	1	63	CML de novo MDS	Completed	Mayo Clinic, USA

## Conclusion and future perspectives

Advances in molecular characterization of AML have provided important information for diagnosis, risk stratification, disease monitoring, and optimization of therapeutic strategies. Novel therapies for AML, including refinements of conventional cytotoxic chemotherapies, genetic and epigenetic targeted drugs, as well as immunotherapies, have significantly improved patient outcomes in recent years [103–105]. Newer generation of TKIs, such as Cabozantinib, Sel24-B489, G-749, AMG 925, TTT-3002, and FF-10101 may overcome disease resistance, and likely will further improve patients’ outcomes [105]. While the genomic complexity and the interplay of the many different molecular abnormalities in AML poses a huge challenge to successful translation into more accurate risk stratification and targeted therapy [18, 104, 105], opportunities do arise; various new agents, such as SMO inhibitors, immune checkpoint inhibitors, metabolic and pro-apoptotic agents, monoclonal or bispecific T-cell engager antibodies, antibody-drug conjugates and chimeric antigen receptor- engineered T cells, have been developed or under investigation as new therapies for AML [106, 107]. Molecularly, targeted therapies have changed the landscape of AML treatment and benefited patients with improved survival and quality of life. Yet, more needs to be done to make our patients live better and longer.

## Data Availability

Not Applicable.

## Abbreviations

ALK: Anaplastic lymphoma kinase
Allo-HSCT: Allogeneic hematopoietic stem cell transplantation
AML: Acute myeloid leukemia
AR: Allele ratio
ASXL1: Additional sex combs-like 1
biCEBPA: Biallelic mutations of CCAAT/enhancer binding protein alpha
CEBPA: CCAAT/enhancer binding protein alpha
CML: Chronic myeloid leukemia
CR: Complete remission
CR1: First complete remission
Cri: Complete remission with incomplete hematologic recovery
DNMT3A: DNA methyltransferase 3A
EFS: Event-free survival
ELN: European Leukemia Net
FLT3: FMS-like tyrosine kinase 3
FLT3-ITD: FLT3 mutations internal tandem duplication
FLT3-TKD: FLT3 mutations: tyrosine kinase domain
GvHD: Graft versus host disease
Hh: Hedgehog
IDH: Isocitrate dehydrogenases
KMT2A: Lysine (K)-specific Methyltransferase 2A
moCEBPA: Monoallelic mutations of CCAAT/enhancer binding protein alpha
MRD: Minimal residual disease
NGS: Next-generation sequencing
NPM1: Nucleophosmin
OS: Overall survival
PDGFR: Platelet-derived growth factor receptors
R/R: Relapsed/refractory
RTK: Receptor tyrosine kinase
RUNX1: Runt-related transcription factor 1
SMO: Smoothened
STAT5: Signal transducer and activator of transcription 5
TKI: Tyrosine kinase inhibitor
TP53: Tumor protein p53
VAF: Variant allele frequency
VEGFR: Vascular endothelial growth factor receptor
WBC: White blood cells
WT1: Wilms’ tumour 1
